# 1-Dichloro­acetyl-*t*-3,*t*-5-dimethyl-*r*-2,*c*-6-diphenyl­piperidin-4-one

**DOI:** 10.1107/S1600536813007927

**Published:** 2013-03-28

**Authors:** P. Sugumar, R. Kayalvizhi, R. Mini, S. Ponnuswamy, M. N. Ponnuswamy

**Affiliations:** a Centre of Advanced Study in Crystallography and Biophysics, University of Madras, Guindy Campus, Chennai 600 025, India; b Department of Chemistry, Government Arts College (Autonomous), Coimbatore 641 018, India

## Abstract

In the title compound, C_21_H_21_Cl_2_NO_2_, the piperidine ring adopts a distorted boat conformation. The phenyl rings substituted at the 2- and 6-positions of the piperidine ring subtend angles of 87.9 (7) and 70.8 (9)°, respectively, with the best plane through the piperidine ring. In the crystal, mol­ecules are connected by C—H⋯O and C—H⋯Cl inter­actions into layers in the *ab* plane.

## Related literature

For the biological activity of piperidine derivatives, see: Aridoss *et al.* (2009 ▶); Michael (2001 ▶); Pinder (1992 ▶); Rubiralta *et al.* (1991 ▶). For puckering parameters, see: Cremer & Pople (1975 ▶). For asymmetry parameters, see: Nardelli (1983 ▶). For hydrogen-bond motifs, see: Bernstein *et al.*(1995 ▶).
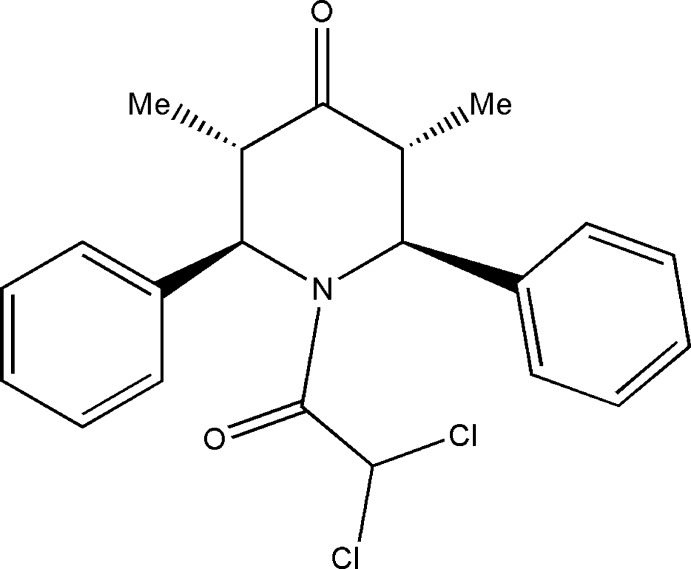


## Experimental

### 

#### Crystal data

C_21_H_21_Cl_2_NO_2_
*M* *_r_* = 390.29
Monoclinic, 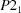

*a* = 8.278 (2) Å
*b* = 9.714 (3) Å
*c* = 11.847 (3) Å
β = 90.578 (9)°
*V* = 952.5 (5) Å^3^
*Z* = 2
Mo *K*α radiation
μ = 0.36 mm^−1^
*T* = 293 K
0.20 × 0.18 × 0.17 mm

#### Data collection

Bruker SMART APEXII CCD diffractometer
Absorption correction: multi-scan (*SADABS*; Bruker, 2008 ▶) *T*_min_ = 0.931, *T*_max_ = 0.944
8874 measured reflections
4241 independent reflections
3962 reflections with *I* > 2σ(*I*)
*R*_int_ = 0.025

#### Refinement

*R*[*F* ^2^ > 2σ(*F* ^2^)] = 0.032
*wR*(*F* ^2^) = 0.088
*S* = 1.03
4241 reflections
235 parameters
1 restraint
H-atom parameters constrained
Δρ_max_ = 0.31 e Å^−3^
Δρ_min_ = −0.31 e Å^−3^
Absolute structure: Flack (1983 ▶), 1745 Friedel pairs
Flack parameter: 0.01 (5)

### 

Data collection: *APEX2* (Bruker, 2008 ▶); cell refinement: *SAINT* (Bruker, 2008 ▶); data reduction: *SAINT*; program(s) used to solve structure: *SHELXS97* (Sheldrick, 2008 ▶); program(s) used to refine structure: *SHELXL97* (Sheldrick, 2008 ▶); molecular graphics: *ORTEP-3* for Windows (Farrugia, 2012 ▶); software used to prepare material for publication: *SHELXL97* and *PLATON* (Spek, 2009 ▶).

## Supplementary Material

Crystal structure: contains datablock(s) global, I. DOI: 10.1107/S1600536813007927/bt6893sup1.cif

Structure factors: contains datablock(s) I. DOI: 10.1107/S1600536813007927/bt6893Isup2.hkl

Supplementary material file. DOI: 10.1107/S1600536813007927/bt6893Isup3.cml

Additional supplementary materials:  crystallographic information; 3D view; checkCIF report

## Figures and Tables

**Table 1:** Hydrogen-bond geometry (Å, °)

*D*—H⋯*A*	*D*—H	H⋯*A*	*D*⋯*A*	*D*—H⋯*A*
C2—H2⋯O1^i^	0.98	2.45	3.379 (2)	159
C20—H20⋯O1^i^	0.98	2.53	3.273 (2)	132
C21—H21*C*⋯Cl1^ii^	0.96	2.81	3.702 (2)	155

Symmetry codes: (i) 
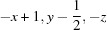
; (ii) 
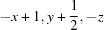
.

**Fig. 1.**
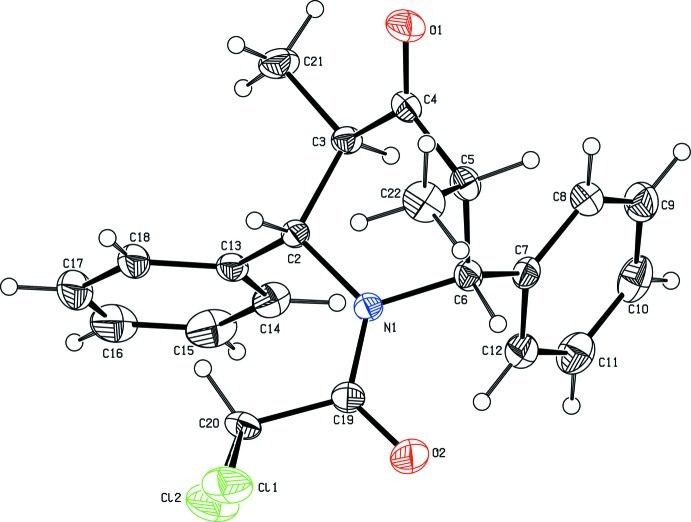
The molecular structure of the title compound, showing the atomic numbering and displacement ellipsoids drawn at 50% probability level.

**Fig. 2.**
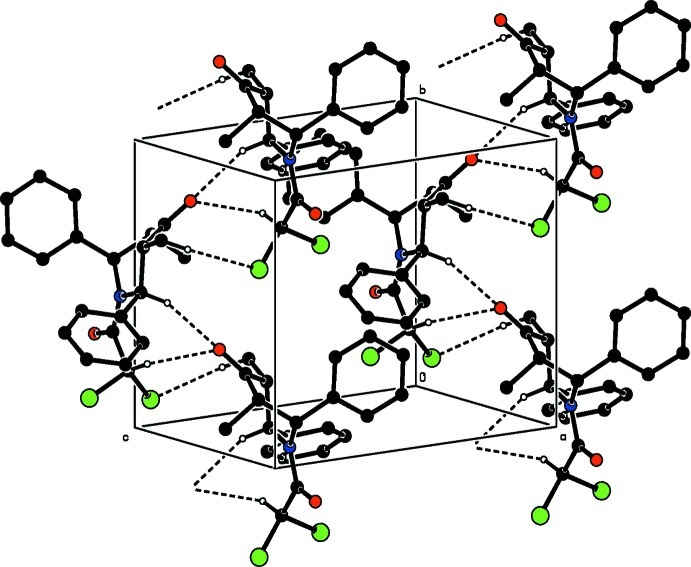
The crystal packing of the molecules. H atoms not involved in hydrogen bonding (dashed lines) have been omitted for clarity.

## supplementary crystallographic information

### Comment

Piperidine derivatives are the valuable heterocyclic compounds in the field of medicinal chemistry. The compounds possessing an amide bond linkage have a wide range of biological activities such as antimicrobial, anti-inflammatory, antiviral, antimalarial and general anesthetics (Aridoss *et al.*, 2009). Functionalized piperidines are familiar substructures found in biologically active natural products and synthetic pharmaceuticals (Michael, 2001; Pinder, 1992; Rubiralta *et al.*, 1991). Against this background and to ascertain the molecular structure and conformation, the X-ray crystal structure determination of the title compound has been carried out.

The *ORTEP* plot of the molecule is shown in Fig. 1. The title compound crystallizes in the monoclinic space group *P*2_1_. The piperidine ring adopts a distorted boat conformation. The puckering parameters (Cremer & Pople, 1975) and the asymmetry parameters (Nardelli, 1983) are: q_2_=0.7556 (2) Å, q_3_ = -0.010 (2) Å, φ_2_ = 287.05 (1)° and Δ_s_(C3 & C6)= 17.08 (1)°. The sum of the bond angles around N1 (359.1°) is in accordance with *sp^2^* hybridization.

The carbonyl group is oriented *syn* to C2 [C2—N1—C7—O1=] -6.5 (2)° and *anti* to C6 [C6—N1—C7—O1=] -176.7 (1)°. The best plane of the piperidine ring and the attached phenyl rings [C7—C12 and C13—C18] enclose dihedral angles of 87.9 (7)° and 70.8 (9)°. The two phenyl rings are oriented to each other with a dihedral angle of 54.01 (1)°.

The crystal packing reveals that the molecules are linked through a network of C—H···O and C—H···Cl intermolecular interactions. Atoms C2 and C20 of the molecule at (*x*, *y*, *z*) donate a proton to bifurcated acceptor atom O1 of the molecule at (1 - *x*,-1/2 + *y*,-*z*), which form two different C(5) and C(8) chains (Bernstein *et al.*, 1995) forming layers in the ab plane as shown in Fig. 2.

### Experimental

t-3,t-5-Dimethyl-r-2,c-6-diphenylpiperidin-4-one (5 mmol) was dissolved in 60 ml of anhydrous benzene. To this solution, dichloroacetylchloride (20 mmol) and triethylamine (20 mmol) were added and the reaction mixture was allowed to stirr for 8 h. The course of the reaction was monitored by TLC. The organic layer was dried over anhydrous Na_2_SO_4_ and the resulting pasty mass was purified by recrystallization from ethyl acetate. Yield: 70%, Melting point: 190–92°C

### Refinement

All H atom were found in a difference map but they were positioned geometrically (C–H = 0.93–0.98 Å) and allowed to ride on their parent atoms, with *U*_iso_(H) = 1.5*U*_eq_(C) for methyl H atoms and 1.2*U*_eq_(C) for all other H atoms.

### Crystal data

**Table:**

C_21_H_21_Cl_2_NO_2_	*F*(000) = 408
*M**_r_* = 390.29	*D*_x_ = 1.361 Mg m^−^^3^
Monoclinic, *P*2_1_	Mo *K*α radiation, λ = 0.71073 Å
Hall symbol: P 2yb	Cell parameters from 3962 reflections
*a* = 8.278 (2) Å	θ = 1.7–28.4°
*b* = 9.714 (3) Å	µ = 0.36 mm^−^^1^
*c* = 11.847 (3) Å	*T* = 293 K
β = 90.578 (9)°	Block, white crystalline
*V* = 952.5 (5) Å^3^	0.20 × 0.18 × 0.17 mm
*Z* = 2	

### Data collection

**Table:**

Bruker SMART APEXII CCD diffractometer	4241 independent reflections
Radiation source: fine-focus sealed tube	3962 reflections with *I* > 2σ(*I*)
Graphite monochromator	*R*_int_ = 0.025
ω and φ scans	θ_max_ = 28.4°, θ_min_ = 1.7°
Absorption correction: multi-scan (*SADABS*; Bruker, 2008)	*h* = −10→10
*T*_min_ = 0.931, *T*_max_ = 0.944	*k* = −11→12
8874 measured reflections	*l* = −15→15

### Refinement

**Table:**

Refinement on *F*^2^	Secondary atom site location: difference Fourier map
Least-squares matrix: full	Hydrogen site location: inferred from neighbouring sites
*R*[*F*^2^ > 2σ(*F*^2^)] = 0.032	H-atom parameters constrained
*wR*(*F*^2^) = 0.088	*w* = 1/[σ^2^(*F*_o_^2^) + (0.0442*P*)^2^ + 0.1557*P*] where *P* = (*F*_o_^2^ + 2*F*_c_^2^)/3
*S* = 1.03	(Δ/σ)_max_ < 0.001
4241 reflections	Δρ_max_ = 0.31 e Å^−^^3^
235 parameters	Δρ_min_ = −0.31 e Å^−^^3^
1 restraint	Absolute structure: Flack (1983), 1745 Friedel pairs
Primary atom site location: structure-invariant direct methods	Flack parameter: 0.01 (5)

### Special details

**Table:**

Geometry. All e.s.d.'s (except the e.s.d. in the dihedral angle between two l.s. planes) are estimated using the full covariance matrix. The cell e.s.d.'s are taken into account individually in the estimation of e.s.d.'s in distances, angles and torsion angles; correlations between e.s.d.'s in cell parameters are only used when they are defined by crystal symmetry. An approximate (isotropic) treatment of cell e.s.d.'s is used for estimating e.s.d.'s involving l.s. planes.
Refinement. Refinement of *F*^2^ against ALL reflections. The weighted *R*-factor *wR* and goodness of fit *S* are based on *F*^2^, conventional *R*-factors *R* are based on *F*, with *F* set to zero for negative *F*^2^. The threshold expression of *F*^2^ > σ(*F*^2^) is used only for calculating *R*-factors(gt) *etc*. and is not relevant to the choice of reflections for refinement. *R*-factors based on *F*^2^ are statistically about twice as large as those based on *F*, and *R*- factors based on ALL data will be even larger.

### Fractional atomic coordinates and isotropic or equivalent isotropic displacement parameters (Å^2^)

**Table:**

	*x*	*y*	*z*	*U*_iso_*/*U*_eq_	
C2	0.41560 (17)	0.55485 (16)	0.18606 (12)	0.0275 (3)	
H2	0.4759	0.5181	0.1220	0.033*	
C3	0.42988 (17)	0.71381 (17)	0.18432 (12)	0.0298 (3)	
H3	0.3622	0.7493	0.2452	0.036*	
C4	0.35940 (19)	0.76681 (17)	0.07391 (12)	0.0321 (3)	
C5	0.19801 (19)	0.7045 (2)	0.03920 (12)	0.0349 (3)	
H5	0.1242	0.7795	0.0186	0.042*	
C6	0.12278 (18)	0.62258 (17)	0.13576 (13)	0.0315 (3)	
H6	0.0301	0.5735	0.1030	0.038*	
C7	0.05790 (17)	0.70465 (19)	0.23542 (12)	0.0332 (3)	
C8	0.0412 (2)	0.8464 (2)	0.23415 (16)	0.0434 (4)	
H8	0.0746	0.8959	0.1714	0.052*	
C9	−0.0246 (2)	0.9155 (2)	0.32501 (19)	0.0522 (5)	
H9	−0.0357	1.0107	0.3226	0.063*	
C10	−0.0736 (2)	0.8441 (3)	0.41870 (17)	0.0537 (5)	
H10	−0.1167	0.8908	0.4800	0.064*	
C11	−0.0585 (2)	0.7033 (3)	0.42115 (16)	0.0523 (5)	
H11	−0.0920	0.6545	0.4843	0.063*	
C12	0.0064 (2)	0.6335 (2)	0.33014 (15)	0.0425 (4)	
H12	0.0155	0.5381	0.3326	0.051*	
C13	0.49049 (18)	0.49813 (17)	0.29406 (13)	0.0328 (3)	
C14	0.4205 (2)	0.5223 (2)	0.39840 (14)	0.0430 (4)	
H14	0.3252	0.5726	0.4032	0.052*	
C15	0.4943 (3)	0.4706 (3)	0.49568 (18)	0.0602 (6)	
H15	0.4470	0.4852	0.5655	0.072*	
C16	0.6365 (3)	0.3980 (3)	0.4893 (2)	0.0650 (7)	
H16	0.6850	0.3642	0.5548	0.078*	
C17	0.7073 (3)	0.3752 (3)	0.3867 (2)	0.0596 (6)	
H17	0.8035	0.3259	0.3828	0.071*	
C18	0.6351 (2)	0.4259 (2)	0.28862 (17)	0.0424 (4)	
H18	0.6838	0.4115	0.2192	0.051*	
C19	0.1845 (2)	0.38367 (19)	0.17034 (15)	0.0373 (3)	
C20	0.3085 (2)	0.26688 (18)	0.17704 (14)	0.0373 (3)	
H20	0.4172	0.3033	0.1639	0.045*	
C21	0.6019 (2)	0.7649 (2)	0.20659 (16)	0.0467 (4)	
H21A	0.6406	0.7286	0.2772	0.070*	
H21B	0.6022	0.8637	0.2097	0.070*	
H21C	0.6709	0.7346	0.1469	0.070*	
C22	0.2237 (3)	0.6139 (2)	−0.06545 (14)	0.0496 (5)	
H22A	0.1226	0.5737	−0.0883	0.074*	
H22B	0.2995	0.5422	−0.0476	0.074*	
H22C	0.2649	0.6693	−0.1258	0.074*	
N1	0.24080 (15)	0.51501 (14)	0.17151 (10)	0.0289 (3)	
O1	0.42761 (17)	0.84953 (16)	0.01538 (11)	0.0479 (3)	
O2	0.04081 (17)	0.35524 (17)	0.16311 (17)	0.0645 (4)	
Cl1	0.25867 (8)	0.14398 (5)	0.07228 (5)	0.06394 (16)	
Cl2	0.30062 (9)	0.18963 (7)	0.31158 (5)	0.07162 (19)	

### Atomic displacement parameters (Å^2^)

**Table:**

	*U*^11^	*U*^22^	*U*^33^	*U*^12^	*U*^13^	*U*^23^
C2	0.0262 (6)	0.0266 (8)	0.0297 (6)	−0.0013 (5)	−0.0018 (5)	0.0000 (5)
C3	0.0304 (7)	0.0279 (8)	0.0310 (6)	−0.0042 (6)	−0.0020 (5)	0.0007 (6)
C4	0.0377 (8)	0.0263 (8)	0.0324 (7)	0.0037 (6)	0.0017 (6)	0.0005 (6)
C5	0.0378 (7)	0.0354 (9)	0.0315 (7)	0.0045 (7)	−0.0055 (6)	0.0027 (6)
C6	0.0287 (7)	0.0307 (8)	0.0349 (7)	0.0009 (6)	−0.0062 (5)	−0.0001 (6)
C7	0.0242 (6)	0.0358 (8)	0.0395 (7)	0.0013 (6)	−0.0021 (5)	−0.0009 (7)
C8	0.0412 (8)	0.0362 (10)	0.0530 (10)	0.0021 (7)	0.0023 (7)	0.0015 (8)
C9	0.0440 (10)	0.0418 (12)	0.0707 (13)	0.0046 (8)	0.0006 (9)	−0.0132 (10)
C10	0.0371 (9)	0.0712 (15)	0.0527 (11)	0.0061 (9)	0.0005 (7)	−0.0208 (10)
C11	0.0452 (9)	0.0680 (14)	0.0440 (9)	0.0016 (10)	0.0077 (7)	−0.0004 (10)
C12	0.0377 (8)	0.0437 (10)	0.0461 (8)	0.0023 (7)	0.0044 (6)	0.0034 (8)
C13	0.0331 (7)	0.0290 (8)	0.0360 (7)	−0.0048 (6)	−0.0061 (6)	0.0041 (6)
C14	0.0417 (9)	0.0524 (12)	0.0348 (8)	−0.0064 (8)	−0.0040 (6)	0.0025 (8)
C15	0.0681 (13)	0.0767 (17)	0.0357 (8)	−0.0239 (12)	−0.0115 (8)	0.0103 (9)
C16	0.0658 (14)	0.0669 (16)	0.0615 (13)	−0.0179 (12)	−0.0338 (11)	0.0273 (12)
C17	0.0469 (10)	0.0527 (13)	0.0785 (15)	0.0011 (9)	−0.0264 (10)	0.0176 (11)
C18	0.0376 (8)	0.0378 (10)	0.0517 (9)	0.0007 (7)	−0.0084 (7)	0.0055 (8)
C19	0.0361 (8)	0.0298 (9)	0.0458 (8)	−0.0041 (6)	−0.0025 (6)	−0.0042 (7)
C20	0.0464 (9)	0.0239 (8)	0.0417 (8)	−0.0052 (7)	0.0032 (7)	0.0003 (6)
C21	0.0419 (9)	0.0450 (11)	0.0530 (10)	−0.0171 (8)	−0.0098 (7)	0.0057 (8)
C22	0.0630 (11)	0.0531 (14)	0.0325 (8)	−0.0012 (9)	−0.0049 (7)	−0.0057 (8)
N1	0.0272 (6)	0.0259 (7)	0.0335 (6)	−0.0021 (5)	−0.0038 (4)	−0.0011 (5)
O1	0.0553 (7)	0.0419 (8)	0.0468 (7)	−0.0025 (6)	0.0068 (6)	0.0151 (6)
O2	0.0394 (7)	0.0393 (9)	0.1147 (14)	−0.0121 (6)	−0.0077 (8)	−0.0083 (9)
Cl1	0.0835 (4)	0.0399 (3)	0.0688 (3)	−0.0167 (3)	0.0168 (3)	−0.0214 (2)
Cl2	0.0933 (4)	0.0638 (4)	0.0576 (3)	−0.0154 (3)	−0.0061 (3)	0.0249 (3)

### Geometric parameters (Å, º)

**Table:**

C2—N1	1.5060 (19)	C12—H12	0.9300
C2—C13	1.519 (2)	C13—C18	1.390 (2)
C2—C3	1.549 (2)	C13—C14	1.390 (2)
C2—H2	0.9800	C14—C15	1.393 (3)
C3—C4	1.517 (2)	C14—H14	0.9300
C3—C21	1.528 (2)	C15—C16	1.375 (4)
C3—H3	0.9800	C15—H15	0.9300
C4—O1	1.205 (2)	C16—C17	1.372 (4)
C4—C5	1.520 (2)	C16—H16	0.9300
C5—C6	1.531 (2)	C17—C18	1.392 (3)
C5—C22	1.537 (2)	C17—H17	0.9300
C5—H5	0.9800	C18—H18	0.9300
C6—N1	1.489 (2)	C19—O2	1.224 (2)
C6—C7	1.527 (2)	C19—N1	1.358 (2)
C6—H6	0.9800	C19—C20	1.531 (3)
C7—C8	1.384 (3)	C20—Cl2	1.7634 (18)
C7—C12	1.389 (2)	C20—Cl1	1.7679 (18)
C8—C9	1.385 (3)	C20—H20	0.9800
C8—H8	0.9300	C21—H21A	0.9600
C9—C10	1.373 (3)	C21—H21B	0.9600
C9—H9	0.9300	C21—H21C	0.9600
C10—C11	1.373 (4)	C22—H22A	0.9600
C10—H10	0.9300	C22—H22B	0.9600
C11—C12	1.387 (3)	C22—H22C	0.9600
C11—H11	0.9300		
			
N1—C2—C13	112.74 (12)	C7—C12—H12	119.7
N1—C2—C3	109.15 (12)	C18—C13—C14	119.57 (15)
C13—C2—C3	110.01 (12)	C18—C13—C2	119.23 (14)
N1—C2—H2	108.3	C14—C13—C2	121.15 (15)
C13—C2—H2	108.3	C13—C14—C15	119.4 (2)
C3—C2—H2	108.3	C13—C14—H14	120.3
C4—C3—C21	112.84 (14)	C15—C14—H14	120.3
C4—C3—C2	108.73 (12)	C16—C15—C14	120.5 (2)
C21—C3—C2	113.12 (14)	C16—C15—H15	119.7
C4—C3—H3	107.3	C14—C15—H15	119.7
C21—C3—H3	107.3	C17—C16—C15	120.35 (18)
C2—C3—H3	107.3	C17—C16—H16	119.8
O1—C4—C3	122.95 (15)	C15—C16—H16	119.8
O1—C4—C5	121.70 (15)	C16—C17—C18	119.9 (2)
C3—C4—C5	115.32 (13)	C16—C17—H17	120.1
C4—C5—C6	111.58 (12)	C18—C17—H17	120.1
C4—C5—C22	108.55 (14)	C13—C18—C17	120.20 (19)
C6—C5—C22	111.46 (16)	C13—C18—H18	119.9
C4—C5—H5	108.4	C17—C18—H18	119.9
C6—C5—H5	108.4	O2—C19—N1	123.08 (17)
C22—C5—H5	108.4	O2—C19—C20	119.15 (17)
N1—C6—C7	112.41 (12)	N1—C19—C20	117.76 (14)
N1—C6—C5	107.86 (12)	C19—C20—Cl2	109.33 (12)
C7—C6—C5	117.09 (14)	C19—C20—Cl1	108.19 (12)
N1—C6—H6	106.3	Cl2—C20—Cl1	109.67 (10)
C7—C6—H6	106.3	C19—C20—H20	109.9
C5—C6—H6	106.3	Cl2—C20—H20	109.9
C8—C7—C12	118.20 (17)	Cl1—C20—H20	109.9
C8—C7—C6	123.16 (15)	C3—C21—H21A	109.5
C12—C7—C6	118.58 (17)	C3—C21—H21B	109.5
C7—C8—C9	120.92 (18)	H21A—C21—H21B	109.5
C7—C8—H8	119.5	C3—C21—H21C	109.5
C9—C8—H8	119.5	H21A—C21—H21C	109.5
C10—C9—C8	120.4 (2)	H21B—C21—H21C	109.5
C10—C9—H9	119.8	C5—C22—H22A	109.5
C8—C9—H9	119.8	C5—C22—H22B	109.5
C9—C10—C11	119.47 (19)	H22A—C22—H22B	109.5
C9—C10—H10	120.3	C5—C22—H22C	109.5
C11—C10—H10	120.3	H22A—C22—H22C	109.5
C10—C11—C12	120.4 (2)	H22B—C22—H22C	109.5
C10—C11—H11	119.8	C19—N1—C6	115.62 (13)
C12—C11—H11	119.8	C19—N1—C2	124.85 (13)
C11—C12—C7	120.6 (2)	C6—N1—C2	118.62 (12)
C11—C12—H12	119.7		
			
N1—C2—C3—C4	−57.74 (15)	N1—C2—C13—C18	128.73 (16)
C13—C2—C3—C4	178.07 (11)	C3—C2—C13—C18	−109.19 (17)
N1—C2—C3—C21	176.08 (12)	N1—C2—C13—C14	−54.1 (2)
C13—C2—C3—C21	51.89 (17)	C3—C2—C13—C14	68.04 (19)
C21—C3—C4—O1	−5.9 (2)	C18—C13—C14—C15	−1.7 (3)
C2—C3—C4—O1	−132.20 (17)	C2—C13—C14—C15	−178.90 (18)
C21—C3—C4—C5	172.05 (15)	C13—C14—C15—C16	1.0 (3)
C2—C3—C4—C5	45.70 (17)	C14—C15—C16—C17	−0.2 (4)
O1—C4—C5—C6	−170.08 (16)	C15—C16—C17—C18	0.1 (4)
C3—C4—C5—C6	12.0 (2)	C14—C13—C18—C17	1.6 (3)
O1—C4—C5—C22	66.7 (2)	C2—C13—C18—C17	178.85 (18)
C3—C4—C5—C22	−111.23 (16)	C16—C17—C18—C13	−0.8 (3)
C4—C5—C6—N1	−56.95 (17)	O2—C19—C20—Cl2	−73.1 (2)
C22—C5—C6—N1	64.59 (16)	N1—C19—C20—Cl2	107.58 (16)
C4—C5—C6—C7	70.99 (17)	O2—C19—C20—Cl1	46.2 (2)
C22—C5—C6—C7	−167.47 (14)	N1—C19—C20—Cl1	−133.03 (14)
N1—C6—C7—C8	136.15 (16)	O2—C19—N1—C6	−14.3 (3)
C5—C6—C7—C8	10.4 (2)	C20—C19—N1—C6	164.98 (13)
N1—C6—C7—C12	−46.81 (19)	O2—C19—N1—C2	176.81 (17)
C5—C6—C7—C12	−172.51 (14)	C20—C19—N1—C2	−3.9 (2)
C12—C7—C8—C9	0.2 (3)	C7—C6—N1—C19	104.61 (16)
C6—C7—C8—C9	177.28 (16)	C5—C6—N1—C19	−124.81 (15)
C7—C8—C9—C10	0.4 (3)	C7—C6—N1—C2	−85.74 (16)
C8—C9—C10—C11	−0.7 (3)	C5—C6—N1—C2	44.84 (17)
C9—C10—C11—C12	0.3 (3)	C13—C2—N1—C19	−56.66 (19)
C10—C11—C12—C7	0.4 (3)	C3—C2—N1—C19	−179.22 (14)
C8—C7—C12—C11	−0.6 (3)	C13—C2—N1—C6	134.73 (14)
C6—C7—C12—C11	−177.81 (15)	C3—C2—N1—C6	12.16 (17)

### Hydrogen-bond geometry (Å, º)

**Table:**

*D*—H···*A*	*D*—H	H···*A*	*D*···*A*	*D*—H···*A*
C2—H2···O1^i^	0.98	2.45	3.379 (2)	159
C20—H20···O1^i^	0.98	2.53	3.273 (2)	132
C21—H21*C*···Cl1^ii^	0.96	2.81	3.702 (2)	155

Symmetry codes: (i) −*x*+1, *y*−1/2, −*z*; (ii) −*x*+1, *y*+1/2, −*z*.
